# The Funeral of the American Dental Association

**Published:** 1898-02

**Authors:** G. Alden Mills

**Affiliations:** New York


### THE FUNERAL OF THE AMERICAN DENTAL ASSOCIATION.

BY DR. G. ALDEN MILLS, NEW YORK.

THE Scripture tells us that it is better to go to a funeral than to a house of feasting. The death of the Association is announced. Death, as many look at it, means the end. Nonsense; death is only a change; something dies that a better condition may be evolved. Because this Association ceases it does not follow that it has not generated a progeny that has not enough of good purpose to enact something that will serve a larger good for the coming profession. The election of president at the last session was a gratification, but we think this officer has let a shadow of the funeral fall now that the change has really come.

While the President said much that is recognized as truth regarding the legislative web which has been spun around us, yet it is not feared but that there will be an infusion of energy that will demand a quickly modified state of things throughout the entire union of States, or the whole fabric will be cast to utter destruction. No words are too hot for denunciation of such dastardly domination which stares a noble humanitarian calling in the face as the unjust legislative spirit. It is a menace to all that is good or progressive. No profession can advance against a selfish aristocracy. I marvel that while many have seen this, they have shrugged their shoulders and bent their tender heads to screen themselves from a possible impropriety should they demonstrate the courage of their convictions.

It is none too soon that the schools of education in dentistry are awake to the danger, and a move on their part to throw off the imposed shackles will give a healthy impetus to the whole body. In my opinion such a determination would do more to awaken the latent energies that are so indifferently looking on in face of the cry, "Why do not the societies increase in membership?" The dentists see, just what so many in the lower strata of society see, or imagine they see, that spirit of dominating, selfish power brandishing their bludgeons over them.

While the editor of the INTERNATIONAL DENTAL JOURNAL has, perhaps, unthinkingly permitted much in the editorial of the September number not wholly in accord with my judgment, there is much that is worthy of perusal by all thinking men, and endorse it by bringing their thoughts into active work.

Should there be a united action during the coming year showing a determined purpose to call a halt and say, "Thus far and *no* farther" shall this dominant spirit of the few retard the growth of the majority, who can count among their number many of sincere purpose? I am prepared to declare that the mischief lies between education and arbitrary legislation. There are not a few of other issues, but these would quickly be disposed of could the major issue be met.

The schools are entitled to our respect on general principles. They have done nobly considering all things, and it is believed that at heart the impulses are for the best good of our worthy calling. I would say to the schools, take up the gauntlet and let the mass of our profession see there is a righteous spirit in existence that can only be productive of good. Truly, we are in a crucible, and the real metal will come out of right doing and thinking.

In my view it may prove a providence that there is a degree of uncertainty prevailing concerning the outcome of the newly formed body, the National Dental Association. There are better things ahead for the brave.

